# Dr. Keller, a Russian Physician

**Published:** 1869-04

**Authors:** 


DR. KELLER, a Russian physician, while going his round in a hospital, had just turned his back on a delirious patient, when the latter rose from his bed and struck the doctor on the head with an iron bar, inflicting an injury which proved fatal in seven hours. The patient, who was moribund, was so overcome by the effort that he fell back on the bed and died.

